# Supplementary material for: Are carbon emissions trading and green financial instruments synergistic? -Comprehensive quantitative research based on content analysis
Source: PLoS One. 2024 Mar 7;19(3):e0298601. doi: 10.1371/journal.pone.0298601 (PMC10919601; doi:10.1371/journal.pone.0298601)
Supplement: S1 Data — (ZIP) [file pone.0298601.s001.zip › Data/地级市Z_F工作报告.zip!/┴╔─■/▒╛╧¬╩╨/└┤╫╘╣1⁄2╓┌║┼í╛┬φ┐╦╩2╛▌═°í┐_2012.docx]

各位代表：

　　现在，我代表市人民政府向大会报告工作，请予审议，并请市政协委员和各位列席同志提出意见和建议。

　　一、2011年工作回顾

　　2011年是实施“十二五”规划的第一年。一年来，在省委、省政府和本溪市委的正确领导下，市政府团结带领全市人民，开拓创新，扎实工作，较好地完成了年初确定的各项目标任务，实现了“十二五”良好开局。

　　——经济总量突破千亿元。预计：全市地区生产总值实现1050亿元，同比增长14%。地方财政一般预算收入102.4亿元，增长37.2%。规模以上工业增加值504亿元，增长15%。

　　——发展动力持续增强。全社会固定资产投资完成625亿元，增长50%。社会消费品零售总额226亿元，增长18%。出口创汇19.2亿美元，增长24%。实际利用外资3.55亿美元，增长17.9%。引进内资790亿元，增长84%。金融机构贷款余额680亿元，增长13.3%。

　　——三都五城建设继续推进。重组后的本钢集团在全国企业500强中位列第113位。高新区获批为国家创新型产业集群和辽宁省示范产业集群。国家林业局授予本溪“中国枫叶之都”称号。成功创建省级卫生城市。创建国家环保模范城市规划通过国家环保部评审。创建国家园林城市通过国家住建部专家评审。国家森林城市、中国优秀旅游城市创建成果继续巩固。

　　——生态环境建设取得新成效。单位国内生产总值能耗下降3.77%。二氧化硫、化学需氧量分别减排1082吨和1019吨。环境空气质量二级（良）以上天数达349天。城市环境综合整治考核全省排名第4位。

　　——人民生活进一步改善。城镇居民人均可支配收入19550元，增长16.5%。农民人均纯收入9414元，增长20%。城镇登记失业率控制在3.9%以内。居民消费价格指数为105.2%，低于全省平均水平。

　　一年来，主要抓了以下工作：

　　（一）围绕谋发展，全力推进转方式调结构、抓投资上项目

　　三大主导产业协调发展。国家医药基地新增签约项目57个，累计引进各类项目226个。53户医药企业建成投产，27家研发单位投产运营，314个医药品种正式投产。全年实现销售收入220亿元。国家重大新药创制平台全国排名上升到第5位。首次在中国药都举办国家级生物医药高新技术交易会和健康养生博览会。钢铁深加工产业园区落地项目141个，开工项目46个，竣工投产项目17个。桥北产业园2.4平方公里起步区实现“五通一平”，东风湖产业园10万平方米标准化厂房投入使用。旅游度假产业集聚区建设加快推进。水洞温泉旅游度假区7个功能区招商地块全部签约，“五桥一路”基本完成。汤沟、大川、思山岭等旅游度假区开工项目14个。

　　项目年活动扎实有效。建立了全市项目信息管理系统。全年共推进重点项目788个，其中亿元以上项目227个，10亿元以上项目37个。

　　县区经济发展加快。农业产业化取得成效。桓仁县被省政府命名为酿酒葡萄“一县一业”示范县。永祥绒山羊和张裕葡萄酒项目被省政府确定为投资10亿元以上农产品加工重点项目。县区工业产业集群和服务业集聚区发展定位明晰，两县四区八个工业产业集群入驻企业335户，六个服务业集聚区完成投资74亿元。两县县城和四区新城建设全面推进。全市县区地区生产总值794亿元，财政一般预算收入72.5亿元，分别占全市总量的75.6%和70.8%，成为全市经济增长的重要支撑。

　　工业“五项工程”全面实施。滚动推进了400个工业技改项目。完成并购项目4个。新增规模以上企业100户。重点产业集群实现销售收入500亿元。3户企业完成淘汰落后产能任务。荣获2个中国驰名商标，10个产品成为辽宁名牌，3户企业获得首届市长质量奖。

　　现代农业取得新成果。深入开展“兴农杯”竞赛活动，全市粮食产量30.3万吨，林地经济面积达到435万亩。引进培育超亿元农事龙头企业30户。建成10个现代农业示范区、96个设施农业小区、60个畜禽标准化养殖小区。完成小型农田水利灌溉工程169项。落实万亩滴灌节水工程。

　　服务业发展态势良好。全市房地产开发、旅游度假、交通运输、通讯信息、金融服务、批发零售、餐饮住宿、文化娱乐等服务业加快发展。全年房地产新开工面积600万平方米，竣工面积330万平方米。商品房销售面积320万平方米，销售额140亿元，两项指标增幅居全省前列。成功举办了第八届中国本溪国际枫叶节和东亚旅游交易会。旅游总收入增幅超过30%。“万村千乡市场工程”村级覆盖率87.1%。全市服务业增加值实现336.1亿元，增长14%。

　　（二）围绕优环境，进一步加快幸福宜居城市建设步伐

　　沈本城市一体化取得新进展。沈溪新城公共行政中心、药都博物馆、公安指挥中心、药都医院、高中分校主体竣工，戴斯酒店和药都广场部分投入使用。实施了药都大街、电力工程等62个基础设施建设项目。11个村的居民回迁保障房全面启动。俊安、旺力、绿地、荣盛、明华等一批房地产开发企业签约入驻。本溪主城规划建设“十里金街”，推进建设商业、商务、文化和住宅地产项目37个。在主城区西部规划建设桥北新城，签约引进城市开发和基础设施项目13个。在主城区东部建设太子河新城，绿地、佳兆业、恒大等一批国内百强地产企业项目开工建设。进一步加强主城区市容管理。城区栽植树木46万株，新增改造绿地75.6万平方米。中心城区道路清扫保洁率不断提升。以“五化”工程为重点的城市环境综合整治取得明显成效。

　　基础设施不断完善。通灌铁路桓仁段主体工程完工。丹通、桓永高速公路桓仁段进入路面施工阶段。沈丹客运专线完成工程量的32%。沈本产业大道全线竣工通车。钢铁大道完成路基桥梁和阿家岭隧道工程。太子河景观路完成卧龙大桥、三家子大桥、同江峪互通立交等桥梁及部分连接线建设。育龙路达到通车条件。维修改造了滨河南路等18条市政道路。建设二电新热源等五项热源，改造了94台锅炉、34座换热站和近10万延长米老旧管网，初步解决了4万多户居民多年低温困扰问题。电力、给排水、燃气、通信等基础设施工程加快建设。

　　节能减排环境治理进一步推进。启动了国际生态安全示范城市申报工作。实施节能减排项目50项。完成15个乡镇、118个村的环境连片整治示范工程，完成农村改厕项目7000座。实施了北沙河综合治理等河道整治工程。在沈本高速、本桓公路等重点路段两侧开展生态环境治理。扎实推进森林“七进”活动，完成人工造林13万亩。

　　（三）围绕增活力，不断加大改革创新和开放招商工作力度

　　本钢重组稳步推进。本钢集团充分发挥整体优势，加速产业升级改造，企业经营取得较好业绩。预计全年钢产量1667万吨，增长3.1%；实现销售收入919亿元，增长20.8%。

　　沈本一体化综合配套改革试点全面启动。本溪生物医药战略性新兴产业、中国药都科技创新体系建设、高新区城乡一体化发展和南芬循环经济区确定为先行先试四项改革试点。加速推进了沈本之间的金融服务、工商管理、社会保障等体制机制一体化。《本溪市城乡统筹综合配套改革试点工作总体方案》深入实施。

　　扩权强县（区）深入实施。市委、市政府制定印发了《关于推进工业产业集群加快发展实施意见》。进一步明确了产业园区体制机制、用人制度、管理权限等扩权强县（区）重点内容。同时明确了各县区事权、财权和责任相统一的管理要求。

　　资金、土地等要素市场创新取得一定成效。银行贷款、发行债券、中期票据、BT融资、企业上市等融资方式，推动了一批项目的实施。通过城乡建设用地增减挂钩等措施，完成新增建设用地报卷900公顷。

　　对外开放不断开创新局面。采取主题招商、专业招商等多种方式，组织赴美国、香港、温州、重庆、宁波、南京等国家和地区开展招商活动10余次。签约引进各类项目603个，亿元以上项目245个。全市进出口总额占地区生产总值的25.6%，位列全省第3位。

　　（四）围绕惠民生，积极推动社会事业发展和谐社会建设

　　十大民生工程基本完成。实现5.7万人稳定就业。全面启动了城乡居民社会养老保险。为7797名被征地农民落实了养老保险政策。实现了城乡居民医疗保险全覆盖。解决了1.7万名困难企业在职职工的基本医保问题。企业退休人员养老金月增加135元，失业保险金月增加120元，市区和两县最低工资标准月提高150元和130元。城乡低保标准分别提高了12.3%和10.7%。全市形成了遍布城乡的法律援助1小时服务圈。新建和改扩建幼儿园和养老服务机构24家。在全省率先建成两所农村常年病人托管中心。完成43个旧住宅小区和主城区114条“摸黑路”整治任务。实施11片国有工矿棚户区改造工程。基本完成2.8万套保障性住房建设。建成4.5万个社会治安动态视频监控点。新建82处农村饮水安全工程。市民关注的出租车计价器全面启用。解决了2.5万户历史遗留房屋产权登记问题。

　　各项社会事业又有新进步。理顺了城区初中管理体制。市机电工程学校一期工程主体完工。成立了市教育基金会。国有文艺院团转企改制稳步推进。举办了本溪首届“红歌大赛”、首届文化产业博览会。电视剧《情暖万家》、话剧《民意如天》分别在央视一套黄金时段播出和进京及全省巡回演出，引起反响受到好评。中国地质博物馆本溪馆正式挂牌。完成市中心医院门诊楼和7所社区卫生服务中心建设。举办了全国竞走大奖赛和本溪市首届体育节。CBA联赛辽宁队主场首次落户本溪。“十二运”分赛场沈溪新城体育中心开工建设。完成第六次全国人口普查任务，扎实推进企业一套表改革工作。全市人口自然增长率控制在0.78‰。实现全国双拥模范城“七连冠”。获得全国法制宣传教育先进城市“三连冠”。档案工作获全国档案系统先进单位。人防工作被沈阳军区评为先进单位。成功举办首届残疾人文化艺术节。完成第八届城市居委会换届选举。民族宗教、外事侨务、国家安全、对台、质监、药监、地方志、优抚、老龄、地震、气象等工作取得新成绩。

　　社会保持基本稳定。扎实开展信访工作效率年活动，深化领导包案和领导干部大接访制度，有效化解了一批突出信访问题和隐患矛盾。加大巡逻防控、打击犯罪、治安整治和公共安全管理力度，全市治安局面持续稳定。社区矫正工作全面铺开，实现了刑释解教人员的无缝对接。不间断地开展安全生产隐患大排查、大整治和“打非治违”专项行动，全市安全生产形势有所好转。

　　法治政府建设有效推进。自觉接受人大和政协监督，认真办理人大代表建议和政协提案958件，办复率100%，解决率69.3%，满意率99.3%。坚持依法行政，提请市人大常委会制定地方性法规４件，制定和修改政府规章5件，清理政府规章88件。落实政府系统党风廉政建设责任制，启动了公共资源交易系统等“五大系统”建设。加强行政监察和审计监督。实施信访稳定、服务环境和县区发展绩效考评工作，促进了政府职能转变和作风改进。

　　各位代表，上述成绩来之不易。这是市委把握全局、正确领导的结果，是市人大和市政协监督支持、共同努力的结果，是全市人民齐心协力、团结奋斗的结果。在此，我代表本溪市人民政府，向辛勤工作在全市各条战线上的广大工人、农民、知识分子、干部群众，向驻溪部队、武警官兵和公安干警，向各民主党派、无党派人士、工商联、各人民团体，向所有关心、热爱、支持和参与本溪发展的各界朋友，表示衷心的感谢和崇高的敬意!

　　在肯定成绩的同时，我们也清醒认识到，当前经济社会发展中还存在一些矛盾和问题：全市经济总量仍然偏小，特别是对产业发展具有牵动作用的大项目数量较少；转方式调结构任务艰巨，主导产业集群尚未形成较强的带动效应；土地、资金、人才等生产要素仍然制约着经济加快发展；财政收支矛盾仍然比较突出；社会稳定压力较大；发展环境有待进一步改善等等。针对这些问题，我们一定要以负责任的态度，高度重视并认真加以解决。

　　二、2012年主要工作任务

　　今年是“十二五”规划全面推进和重点突破之年，抓好全年工作意义重大。当前，国际国内经济环境存在很大的不确定性，经济增长下行的压力加大，新的一年将面临和迎接更加严峻的挑战。我们要辩证地看待当前形势，始终坚持科学发展观，坚定“稳中求进”的自觉性，增强“稳中求快”的紧迫感，强化“能快则快”的赶超意识，全力抓好各方面工作，为“十二五”乃至今后长远发展打下坚实基础。

　　今年政府工作的总体要求是：全面贯彻落实省市委十一届二次全会和省市经济工作会议精神，围绕“昂首十二五、建设新本溪”的总体目标，坚持稳中求进，落实稳中求快，加快推进沈本一体化，加快建设“三都五城”，更加关注项目建设，更加关注实体经济，更加关注城乡统筹，更加关注县区发展，更加关注改革开放，更加关注民生改善，推动全市经济社会实现科学发展、创新发展、和谐发展。

　　全市经济社会发展的主要预期和控制目标是：地区生产总值增长13%，地方财政一般预算收入增长15%，规模以上工业增加值增长15%，全社会固定资产投资增长28%，社会消费品零售总额增长16%，实际利用外资增长30%，出口创汇增长15%，引进内资增长20%，城镇居民人均可支配收入增长14%，农民人均纯收入增长16%，单位国内生产总值能耗下降3.77%，城镇登记失业率控制在4.2%以内，居民消费价格指数低于全省平均水平。

　　新的一年，市政府要重点做好以下工作：

　　（一）以项目建设为龙头，全力保持投资较快增长

　　把增投资、上项目作为关系本溪长远发展的战略举措，努力让更多的项目扎根本溪、更多的资金涌入本溪。深入开展项目年活动。坚持市、县区领导包项目责任制，按照部门工作项目化的要求，以县区和部门为单位制定项目计划，加大项目调度、通报和督察力度，努力形成储备一批、开工一批、投产一批的项目运行机制。切实抓好基础设施、新型工业、房地产开发、旅游业、社会事业、现代农业和生态环境等重点领域项目的投资建设，全力推进一批亿元特别是10亿元以上的重大项目。加快本溪电网建设。开工建设观音阁水库输水工程。推进沈丹客运专线加快建设。确保桓仁“两高一铁”、钢铁大道、太子河景观路等项目竣工通车。启动建设304国道千金岭隧道。

　　（二）以结构调整为主线，不断推动工业经济提速增效

　　通过调整产业结构，尽快建立以主导产业为支柱、工业产业集群为平台、重点企业为支撑的新型工业化发展格局。

　　全力推动钢铁产业加快做强做大。举全市之力支持本钢落实“十二五”规划目标。推进本钢三冷轧、轧辊等项目开工建设，推进北台厂区新1号、2号高炉等项目加快建设，支持本钢上项目、上水平，加快向做优做强方向迈进。依托本钢大力发展钢铁深加工产业，完善桥北、东风湖、南芬等钢铁深加工产业园区基础设施，推动大台沟铁矿、思山岭铁矿、聚鑫达矿业、金永新材冷轧板等重点项目加快建设。

　　加快发展生物医药产业。全力推动投产企业达产达效、开工企业竣工投产、签约企业开工建设，力争投产运营企业达到69户、研发机构达到34个。全力抓好“百强企业”、“百人计划”、标准化厂房项目的签约落地，落实好相关扶持政策，引进知名药业企业10家以上、世界一流的海外研发团队30个以上，不断提升中国药都的项目质量和国际化水平。全年销售收入要实现300亿元。

　　大力培育县区工业产业集群。各县区要按照有发展空间、有主导产品、有龙头企业、有产业链等要求，创造环境，加快园区基础设施和标准化厂房建设，每个产业集群都要启动建设50万平方米的标准化厂房；下力气招商引资，每个产业集群都要举办一个高水平的招商洽谈会，以主导产业为重点，引进一批延长产业链、具有核心竞争力的企业和企业总部；下功夫搭建各种配套服务平台，为入驻企业提供研发、金融、人力、物流等服务保障。

　　深入实施工业“五项工程”。全年新增销售收入5亿元以上企业20户，新增规模企业100户以上。抓好重点工业项目建设，力争300个项目开工，100个项目竣工投产，实现工业固定资产投资400亿元。完成并购项目4个。重点产业集群实现销售收入600亿元。确保万元工业增加值能耗年均降幅5.1%，完成省下达的企业淘汰落后产能任务。

　　（三）以“五年翻两番”为目标，加快发展县区经济

　　县域经济要突出“三化”建设。农业产业化要以“一县一业”为重点，本溪县要新发展以林下参为主的中药材10万亩，并申报成为省级“一县一业”示范县；桓仁县要提高酿酒葡萄“一县一业”的水平。培育并推进4个10亿元以上农业产业化龙头企业建设。以促进农民增收为目标，大力发展果品、食用菌、山野菜、优质米等特色产业和高效设施农业。全面落实中央支农惠农政策，积极发展农民专业合作社，千方百计增加农民收入。工业集群化要以产业集群建设为重点，加快推动本溪县传动装置和玻璃制品产业、桓仁县葡萄酒和包装印刷产业的项目集聚和开工建设，完善园区基础设施和体制机制。城镇规模化要以将观音阁新城和五女山新城建设成为30万人口的中等城市为目标，落实好初中进城和放开县城户口两项改革试点任务，进一步完善道路、电力、给排水等基础设施，新增房地产开发面积各120万平方米。加速中心城镇建设，结合土地增减挂钩，将边远山区孤屯散户向城镇搬迁。

　　城区经济要突出“三区”建设。四个城区要抓好工业产业园区建设，积极推动园区内项目集聚、形成规模；抓好特色服务业集聚区建设，重点推动站前中央商贸、卧龙汽车服务、彩北物流和思山岭文化旅游产业园建设；抓好现代化新城区建设，集中精力培育桥北新城、太子河新城、溪湖新城和思山岭新城，完善基础设施、景观环境，推进房地产开发。全市房地产新开工面积要达到700万平方米。推进物流产业发展，为产业集群和园区建设提供物流支撑。积极发展商贸流通业，继续实施“家电下乡”、“万村千乡市场工程”。全年新增批发、零售、住宿、餐饮限额以上法人企业100户。

　　（四）以提升城市软实力为方向，促进文化事业文化产业繁荣发展

　　落实十七届六中全会精神，坚持一手抓公益性文化事业，一手抓经营性文化产业，积极推进以药都文化、钢都文化、山水文化、历史文化和群众文化为主题的文化体系建设，努力建设文化强市。

　　繁荣文化事业。继续推进文化惠民工程，推进艺术宫、书画院及图书馆、档案馆、美术馆、科技馆、创意馆等公共文化基础设施建设，进一步健全覆盖城乡公共文化服务体系。积极推进文化艺术精品力作的繁荣，强化传统文化和非物质文化遗产的传承，加大对文化事业的投入力度和政策支持，实现文化全面发展。

　　发展文化产业。巩固文化体制改革成果，加快国有文艺演出院团转企改制步伐。加快推进本溪湖工业遗址公园、庙后山古人类遗址公园等历史文化项目建设，打造本溪中央文化区、辽砚文化产业园等一批特色文化产业园区，增强本溪文化产业的整体实力和竞争力。设立本溪文化产业投资基金，引导文化产业投资的增加。加强文化产品服务和要素市场建设，提升文化市场管理水平。

　　加快旅游度假产业发展。发挥“中国枫叶之都”品牌优势，大力发展文化旅游、温泉旅游和乡村旅游，加快本溪水洞、温泉寺、汤沟、老边沟、桓龙湖、财神寺以及五女山、铁刹山、关门山等旅游景区的建设步伐。精心培育百家特色旅游山庄，推动本溪由观光型旅游向度假型旅游的转变。完善旅游基础设施建设，着力解决旅游景区“交通难、停车难、住宿难”等问题，提高服务接待水平。积极做好旅游宣传推广，举办好本溪旅游文化节等节庆活动，加快旅游产品和市场开发，全市旅游总收入要保持30%以上增长。

　　（五）以改革开放为动力，进一步增强经济发展活力

　　推动新型工业化综合配套改革。按照沈阳经济区综合配套改革总体部署，选择重点领域和关键环节先行先试。努力将高新区打造成战略性新兴产业示范区，建设国内一流的生物医药产业科技创新体系，加快推进城乡一体化配套改革，加快南芬循环经济区和溪湖再生资源产业园建设步伐。

　　继续深化扩权强县（区）工作。结合我市实际，将扩权改革落实到市级以上工业产业集群和服务业集聚区，在各个产业园区健全管理体制，完善区划调整，下放管理权限，制定优惠政策，推动集群发展。

　　拓展融资方式。加强银企对接，新增贷款100亿元以上。积极发行城投债、企业债和中小企业集合债。全面推动BT、BOT及基金投资。努力争取国家投资。利用好国家开发银行、农发行等政策性银行贷款。推动大企业发行票据融资。全力推动华兴、龙宝、工具、矿业等4户企业加快上市。

　　切实解决土地供给。强化集约节约用地理念，用好新增建设用地指标。积极搞好城乡建设用地增减挂钩。全力做好未利用地、废弃地置换建设用地工作。启动旧村屯、旧社区、旧工厂改造试点。完善土地收购储备程序，推进净地出让。加强低效土地的合理利用。依法依规加大城市旧房屋征收和农村集体土地征用工作力度。

　　构筑区域自主创新体系。加大对科技创新的支持力度，鼓励企业自主创新和研发新产品，培育15家市级以上高新技术企业，建设10家市级企业技术研发中心。引进培育生物医药科技领军人才和产业领军人才20人以上。结合药都大学城等项目建设，打造高素质人力资源培育基地。实施质量兴市和商标战略，引导企业争创知名品牌，提高竞争力。

　　放手发展民营经济。实行非禁即入的准入政策，鼓励民营企业进入国有退出的领域和新兴行业。健全资金、土地、技术、人才、优惠政策等方面服务体系，努力创造各类市场主体平等竞争发展的局面。加快制定中小企业和微型企业发展扶持政策。进一步推动和鼓励自主创业，营造全民创业、竞相发展的浓厚氛围，全市民营经济实现增加值增长25%以上。

　　强力推动开放招商。坚持“走出去、请进来”，围绕工业产业集群、服务业集聚区的发展定位和新城新市镇建设重点，到北美、欧洲等地区进行境外专题招商，到广东、福建、江苏等地进行国内专题招商。全面落实各项优惠政策，重点承接和引进一批世界500强、行业100强企业和产业层次高、投资规模大、带动能力强的内外资项目。跟踪落实已签约项目，不断提高引进项目的合同履约率、资金到位率、项目开工率和投入产出率。鼓励外出劳务和企业利用境外资源投资办厂。努力拓宽出口领域，增加出口产品种类，不断提升本溪企业在国际市场的竞争力。

　　（六）以幸福宜居城市建设为载体，加快推进城市化进程

　　围绕打造健康幸福宜居城市，进一步推动沈本一体化、城乡一体化进程，提升城市化水平。

　　加快建设沈溪新城。要以市级政务中心主体工程建设为重点，加快推进本溪中央文化区建设步伐。以文化中心、体育中心、金融中心、会展中心等为重点，加快完善城市公共服务设施。以沈阳药科大学、中国医科大学、辽宁中医药大学、辽宁医药化工职业技术学院为重点，加快大学城建设。以土地整理、道路、电力、给排水、供热、燃气等为重点，加快完善城市基础设施。以路网绿化、水系景观、山体公园、矿山整治为重点，加快城市景观环境建设。以商业、商务、工业、文化、住宅等地产为重点，加大房地产开发力度。

　　改造提升本溪主城。继续打造好“十里金街”。加快太子河新城基础设施、保障房建设，推进绿地中央广场、佳兆业水岸新都、恒大绿洲等重点商业、住宅地产项目建设进度。全力推进桥北新城的基础设施、景观环境和保障房项目的开发建设。按照创建国家园林城市、国家卫生城市工作要求，加强城市建设和管理。建设北地跨线桥，改造环山路、东芬转盘、铁路转盘及永丰立交桥、彩北两洞桥，形成城市环路，提升城市主干路网功能。城区绿化栽植树木25万株。加快停车设施建设。继续开展以“五化”工程为重点的城市环境综合整治活动。

　　加强生态环境建设。实施“青山、碧水、蓝天”工程。青山工程重点是以“青山杯”竞赛为载体，在“三区两带一企”大力实施矿山植被恢复、小开荒治理等十大工程，完成省下达年度矿山治理任务。碧水工程重点是加快建设中国药都北沙河治理工程、浑江城市段景观工程，启动太子河生态环境综合治理工程，用两年左右时间在太子河流域全面消灭5类水体。蓝天工程重点是实施11项污染源治理工程。力争市区空气质量二级（良）以上天数不低于上年水平。完成省级环保模范城市创建任务。

　　（七）以改善民生为根本，不断提升人民群众生活水平

　　继续为群众办好十二件实事：一是完成国有工矿棚户区改造等保障性安居工程年度任务。二是全年实现城镇实名制就业6.5万人。三是继续提高企业退休人员养老金待遇、失业金标准和城乡低保对象救助水平。四是解决“五七工”、“家属工”的养老保险问题。五是基本解决国有和集体困难企业在职职工基本医保问题。六是将全市1.5万名老工伤人员全部纳入工伤保险统筹范围。七是加强教育现代化、信息化建设，实现义务教育学校“班班通”。八是全面实施国家基本药物制度。九是推进热源建设和管网改造。实施“暖房子”工程。十是加强旧住宅区环境综合整治。继续整治“摸黑路”、改造街巷路。十一是建设48处农村饮水安全工程。十二是落实“菜篮子”工程规划，新增菜田6000亩。

　　（八）以社会管理创新为抓手，积极构建和谐社会

　　全面发展各项社会事业。深化素质教育，强化内涵建设，推进均衡发展，科学规划调整全市教育结构布局，整合教育资源。大力发展学前教育。改造城区薄弱学校。推进桓仁县农村初中进城办学的省级试点。推动医药卫生体制改革纵深发展。加强以全科医生和乡村医生为重点的基层医疗卫生队伍建设，启动建设全科医师培训基地和市公共卫生服务中心。深化公立医院改革，推动医疗资源有效整合。做好“十二运”筹备工作，抓好体育中心等场馆建设。落实人口和计生各项奖励政策，建设市人口和家庭公共服务中心。加强残疾人社会保障和社会服务体系建设。全面落实企业一套表改革。强化关心下一代工作。认真做好人防、国家安全、外事侨务、民族宗教、广播电视、对台、档案、地方志、优抚、老龄、地震、气象等各项工作。

　　维护社会和谐稳定。深入开展“六五”普法，加大法律援助和社区矫正工作力度。继续提高社会救助水平。大力发展社会福利和慈善事业。加强军地融合，支持国防建设。争创全国双拥模范城“八连冠”。加强基层政权和社区建设。积极推进精神文明建设。深入开展社会治安综合治理，进一步完善治安巡逻防控体系，严厉打击各种违法犯罪活动。不间断地开展安全生产大检查、隐患排查和专项整治，全面落实安全生产责任制，坚决遏制各类安全事故的发生。强化应急能力建设。做好森林防火和病虫害防治工作。深入开展领导干部大接访活动，努力从源头上预防和减少信访矛盾的发生。加强食品药品安全、重大动物疫情防控和市场价格监测监管，营造安全放心的消费环境。

　　三、切实加强政府自身建设

　　发展的重任、竞争的态势、人民的意愿，对政府自身建设提出了更新、更高、更严的要求。我们将始终带着责任和激情，忠实履行职责和使命，让本溪的发展一年更比一年强，本溪人民的生活一年更比一年好！

　　坚持解放思想，创造性地开展工作。要把解放思想、与时俱进、改革创新的精神贯穿到政府工作的全过程，用创新的思维想问题、办事情，用改革的办法解难题、促发展，以超常规的举措，推进各项重点工作不断取得新成绩。

　　坚持学习研究，提高科学决策水平。切实加强对科学发展、市场经济和国家政策、法律法规的学习研究，提高政策理论水平，增强工作的前瞻性、预见性、针对性。广泛听取各方面意见，集思广益，集中民智，使决策更加科学民主。

　　坚持转变作风，加大工作落实力度。弘扬“药都精神”和“公安精神”，强化服务意识，提高行政效率，多讲怎么办，少讲不能办。强化绩效考评工作，提高政府机关执行能力。强力整顿机关作风，坚决治理庸懒散问题。强化督察工作，严格执行问责制，确保各项工作按照既定部署，一步一步、扎扎实实地落到实处。

　　坚持依法行政，规范行政执法行为。严格依照法定权限和程序履行职责。自觉接受人大的法律监督和政协的民主监督，广泛听取各民主党派、工商联和无党派人士的意见和建议，重视发挥新闻舆论和社会公众的监督作用。认真办理人大代表建议、政协提案。落实好市人大常委会建议案和市政协建议案。推进政务公开。强化审计监督和行政监察，做到有责必问、有错必纠。

　　坚持廉洁自律，建设人民满意政府。以行政权力运行制度等“五大系统”建设为载体，严格落实党风廉政建设责任制，确保权力规范、透明、阳光运行。始终牢记“两个务必”，严格遵守《廉政准则》，堂堂正正做人，清清白白做官，干干净净做事。树立过紧日子的思想，从严控制行政经费和一般性支出，把有限的财力用于经济发展和民生改善，以实际行动和工作业绩，建设廉洁、务实、高效的政府，建设人民满意、人民信赖的政府！

　　各位代表、同志们，站在新起点，再创新优势，实现新跨越，是全市人民的热切期盼，是时代赋予我们的光荣使命。让我们在省委、省政府和本溪市委的正确领导下，坚定信心、锐意创新、奋发进取、扎实工作，确保全年各项目标任务圆满完成，以优异成绩迎接党的十八大胜利召开。
